# Heat Transport in a Spin-Boson Model at Low Temperatures: A Multilayer Multiconfiguration Time-Dependent Hartree Study

**DOI:** 10.3390/e22101099

**Published:** 2020-09-29

**Authors:** Chou-Hsun Yang, Haobin Wang

**Affiliations:** Department of Chemistry, University of Colorado Denver, Denver, CO 80217-3364, USA; chou.yang@ucdenver.edu

**Keywords:** ML-MCTDH, spin-boson, heat transport, quantum dynamics

## Abstract

Extending our previous work, quantum dynamic simulations are performed to study low temperature heat transport in a spin-boson model where a two-level subsystem is coupled to two independent harmonic baths. Multilayer multiconfiguration time-dependent Hartree theory is used to numerically evaluate the thermal flux, for which the bath is represented by hundreds to thousands of modes. The simulation results are compared with the approximate Redfield theory approach, and the physics is analyzed versus different physical parameters.

## 1. Introduction

Electronic and optical processes are usually accompanied by heat generation and transport. Very often, a central task in technology and engineering is to have such transport processes under control. This is particularly useful in the exploration of new nano-size electronic and optical materials, for example, nanoscale molecular junctions where molecules are connected to metal or semiconductor electrodes. Heat transport is crucial for the stability of such junctions, that is, whether the heat generated at such a scale can be released quickly and efficiently is an important characteristic for a potential molecular electronic device [1,2,3,4,5,6]. It has been shown experimentally that the localized Joule heating may induce a substantial temperature increase within a molecule–metal contact due to inefficient heat dissipation [4]. Theoretical modeling and simulation of heat transport at the nanoscale will thus provide valuable insight into the transport mechanisms, as well as offer interpretation of experimental results and help design practical nanoscale electronic devices.

Heat transport through molecular junctions has been modeled by Segal and coworkers [7,8,9] using a system–bath Hamiltonian where a subsystem (representing a junction) is linearly coupled to two heat baths with different temperatures. When the junction is modeled by a harmonic oscillator, the overall problem is trivially solvable. As soon as the junction becomes a two-level system, which can be viewed as a minimal nonlinear model for the junction heat transport, the Hamiltonian becomes the well-known spin-boson model and exhibits rich physics without closed-form solutions. In the weak system–bath coupling regime, Redfield theory [10,11] can be used to provide an approximate description for the heat transport process [7,8,9]. These studies reviewed possible new nano-devices such as a thermal rectifier [7,8], a molecular heat pump [9] and an absorption refrigerator [12,13]. Since charge and heat currents could be coupled, a nonlinear phonon-thermoelectric device was studied [14]. Recently the interference effects in vibrational heat conduction across single-molecule junctions has also been discussed [15].

The weak-coupling assumption in the Redfield theory is, however, often violated in realistic situations. Improved approximations may be developed, e.g., an analog of the Meir–Wingreen formula [16], a nonequilibrium polaron-transformed Redfield equation for bridging the energy transfer from weak to strong coupling regimes [17,18,19,20,21,22], and a full counting statistics combined with the Keldysh nonequilibrium Green’s function, path integral, quantum-classical Liouville equation [23,24,25,26]. These theoretical development offered valuable insights into the heat transport processes.

To gauge the accuracy of these theoretical results it is important to develop numerically exact treatment of the model, which can be used to study stronger coupling regimes and to provide benchmark results for developing approximate theories. In our previous work [27], we have applied the multilayer multiconfiguration time-dependent Hartree (ML-MCTDH) theory [28,29] to study the dynamics of the spin-boson nanojunction model. In contrast to Redfield theory, our previous study revealed a turnover behavior of the heat current with respect to the coupling strength between the two-level system and the heat baths. As a consequence, the optimization of heat transport is possible by choosing an appropriate set of physical parameters. In this work, we extend our simulation to the low temperature regime where quantum effects are more pronounced. Section 2 discusses the model and a few details of implementation of ML-MCTDH for studying heat transport in the spin-boson model. Section 3 presents the results from the ML-MCTDH simulation at low temperature versus several physical parameters. Section 4 concludes.

## 2. Methods

### 2.1. Hamiltonian

As in the previous work [7,8,9,16,27] we use a spin-boson type Hamiltonian where two states of the model molecular junction are coupled to two phonon baths, left (L) and right (R). The overall Hamiltonian has three parts

(1) $$H=H_{S}+H_{B}+H_{SB},$$

where $H_{s}$ describes the two-level subsystem

(2a) $$H_{S}=E_{0}\left|0\rangle \langle 0\right|+E_{1}\left|1\rangle \langle 1\right|=\bar{E}+\frac{\epsilon }{2}\sigma_{z},$$

(2b) $$\bar{E}=\frac{E_{0}+E_{1}}{2},\;\epsilon =E_{0}-E_{1},$$

$H_{B}$ describes the two harmonic baths in mass-weighted coordinates

(3) $$H_{B}=H_{B_{L}}+H_{B_{R}}=\frac{1}{2}\sum_{i_{L}}\left(p_{i_{L}}^{2}+\omega_{i_{L}}^{2}q_{i_{L}}^{2}\right)+\frac{1}{2}\sum_{i_{R}}\left(p_{i_{R}}^{2}+\omega_{i_{R}}^{2}q_{i_{R}}^{2}\right),$$

$H_{SB}$ describes the coupling between the two-level subsystem and the phonon baths

(4) $$H_{SB}=\sigma_{x}\left(\sum_{i_{L}}c_{i_{L}}q_{i_{L}}+\sum_{i_{R}}c_{i_{R}}q_{i_{R}}\right),$$

and $\sigma_{x}$, $\sigma_{z}$ are standard Pauli matrices

(5a) $$\sigma_{x}=\left|0\rangle \langle 1\right|+|1\rangle \langle 0|,$$

(5b) $$\sigma_{z}=\left|0\rangle \langle 0\right|-|1\rangle \langle 1|.$$

In this Hamiltonian, the system–bath coupling is off-diagonal in the system states. A simple transformation can be used to convert it to the more familiar spin-boson form [30]. Using the relation

(6a) $$U^{T}\sigma_{x}U=\sigma_{z},\;U^{T}\sigma_{z}U=\sigma_{x},$$

where

(6b) $$U=\frac{1}{\sqrt{2}}\left(\sigma_{x}+\sigma_{z}\right)=U^{T},$$

$H_{s}$ and $H_{SB}$ can be transformed to

(7) $$U^{T}H_{S}U=\bar{E}+\frac{\epsilon }{2}\sigma_{x},$$

(8) $$U^{T}H_{SB}U=\sigma_{z}\left(\sum_{i_{L}}c_{i_{L}}q_{i_{L}}+\sum_{i_{R}}c_{i_{R}}q_{i_{R}}\right).$$

Thus, $U^{T}HU$ is the Hamiltonian used in the simulation.

The system–bath coupling strength parameters $c_{i}$’s are determined by the bath spectral densities [30]

(9) $$S\left(\omega \right)=\frac{\pi }{2}\sum_{i}\frac{c_{i}^{2}}{\omega_{i}}\delta \left(\omega -\omega_{i}\right).$$

In this model study, we choose identical spectral densities for the left and right bath, which is in an Ohmic form with an exponential cutoff

(10) $$S\left(\omega \right)=\frac{\pi }{2}\alpha \omega \;e^{-\omega /\omega_{c}},$$

where $\omega_{c}$ is the characteristic frequency of the bath and the Kondo parameter α is related to the classical reorganization energy λ (in the context of electron transfer theories [31] using the spin-boson model) via the relation $\lambda =2\alpha \omega_{c}$. In ML-MCTDH simulations the two continuous baths are discretized to a finite number of modes [29], that is, casting the continuous form of Equation (10) to the discrete form in Equation (9). This can be done using several strategies [32,33,34,35] and for a bath with complex glassy spectral densities [33]. The number of bath modes is increased until convergence is achieved, which will be illustrated in the next section.

### 2.2. Calculating the Heat Current

The heat current or thermal flux is defined as the time derivative of the collective total energy for a heat bath. We can consider an idealized situation that at time zero the two baths are brought into contact with the two-level subsystem. We denote the temperatures as $T_{L}$ and $T_{R}$ for the left and right bath, respectively and, without loss of generality, impose the condition that the left bath has a lower temperature, $T_{L}<T_{R}$. As time evolves the energy will start to flow among the two baths and the two-level subsystem. The time-dependent energies of the left and right bath can be defined as (we use atomic units where ℏ=1)

(11) $$\langle H_{L/R}\left(t\right)\rangle =\frac{1}{\mathrm{tr}[\rho ]}\mathrm{tr}\left[\rho \;e^{iHt}H_{L/R}e^{-iHt}\right],$$

where

(12a) $$H_{L}=\frac{1}{2}\sum_{i_{L}}\left(p_{i_{L}}^{2}+\omega_{i_{L}}^{2}q_{i_{L}}^{2}\right)+\sigma_{z}\sum_{i_{L}}c_{i_{L}}q_{i_{L}}=H_{B_{L}}+\sigma_{z}\sum_{i_{L}}c_{i_{L}}q_{i_{L}},$$

(12b) $$H_{R}=\frac{1}{2}\sum_{i_{R}}\left(p_{i_{R}}^{2}+\omega_{i_{R}}^{2}q_{i_{R}}^{2}\right)+\sigma_{z}\sum_{i_{R}}c_{i_{R}}q_{i_{R}}=H_{B_{R}}+\sigma_{z}\sum_{i_{R}}c_{i_{R}}q_{i_{R}},$$

represents the Hamiltonian for the left and right bath, respectively, and ρ is an initial density matrix of the overall system. The specific choice of ρ affects the transient dynamics of the heat transport, but due to the smallness of the subsystem it does not affect the steady-state heat current. So for convenience, ρ is often chosen in a separable form

(13) $$\rho =e^{-\beta_{L}H_{B_{L}}}e^{-\beta_{R}H_{B_{R}}}\rho_{s},$$

where $\beta_{L}=1/k_{B}T_{L}$, $\beta_{R}=1/k_{B}T_{R}$ with $k_{B}$ the Boltzmann constant, and $\rho_{s}$ is an initial density matrix for the two-level subsystem. In this work, we choose $\rho_{s}$ to be the identity operator for the two-level subsystem.

The existence of the system–bath coupling makes the definition of the energy for each bath somewhat ambiguous. Here we have used $H_{L}/H_{R}$ of Equation (12) to define the energy and subsequently the heat current of the left/right bath. On the other hand, the Hamiltonians of the bare baths, $H_{B_{L}}/H_{B_{R}}$ of Equation (3), could also be used

(14) $$\langle H_{B_{L/R}}\left(t\right)\rangle =\frac{1}{\mathrm{tr}[\rho ]}\mathrm{tr}\left[\rho \;e^{iHt}H_{B_{L/R}}e^{-iHt}\right].$$

For simulation purposes, both definitions give the same long time limit of the steady-state heat current [27], defined as the time-derivative of the bath energy. The use of Equation (12) reduces the magnitude of the transient current and is thus preferred in the simulation. Thereby, the heat current is defined as the energy flux of the left or the right bath (note that we have chosen $T_{L}<T_{R}$, so the steady-state energy flow will be from the right to the left)

(15a) $$J_{L}\equiv \lim_{t\to \infty }J_{L}\left(t\right)=\lim_{t\to \infty }\frac{d\langle H_{L}\left(t\right)\rangle }{dt}=\lim_{t\to \infty }\frac{1}{\mathrm{tr}[\rho ]}\mathrm{tr}\left\{\rho \;e^{iHt}i\left[H,H_{L}\right]e^{-iHt}\right\},$$

(15b) $$J_{R}\equiv \lim_{t\to \infty }J_{R}\left(t\right)=-\lim_{t\to \infty }\frac{d\langle H_{R}\left(t\right)\rangle }{dt}=-\lim_{t\to \infty }\frac{1}{\mathrm{tr}[\rho ]}\mathrm{tr}\left\{\rho \;e^{iHt}i\left[H,H_{R}\right]e^{-iHt}\right\}.$$

Based on Equations (1), (7), (8), and (12), the commutators are given as

(16) $$i\left[H,H_{L/R}\right]=-2\sigma_{y}\epsilon \sum_{i_{L/R}}c_{i_{L/R}}q_{i_{L/R}},$$

where $\sigma_{y}$ is the third Pauli matrix

(17) $$\sigma_{y}=i(|0\rangle \langle 1|-|1\rangle \langle 0|).$$

The short time transient behavior of $J_{L}\left(t\right)$ is different from that of $J_{R}\left(t\right)$ [27]. Their average

(18) $$J\equiv \lim_{t\to \infty }J\left(t\right)=\lim_{t\to \infty }\frac{1}{2}\left[J_{L}\left(t\right)+J_{R}\left(t\right)\right],$$

generally minimizes the large transient characteristic [27], and will be used to calculate the heat current in our simulation.

The quantum mechanical trace in the the expressions above is evaluated via Monte Carlo average using an importance sampling technique [29,36,37,38]. Unlike some reduced properties such as the system population or a reduced density matrix for a particular degree of freedom, calculating the heat current involves all degrees of freedom for a bath. The statistical average and the finite-mode representation of the bath sometimes may cause J(t) to oscillate versus time. This is similar to a quantum reactive scattering calculation in the presence of a scattering continuum where, with a finite number of basis functions, an appropriate absorbing boundary condition is added to mimic the correct outgoing Green’s function [39,40,41,42]. In a previous work [27], we employed such a strategy to regularize the heat current at longer time

(19) $$J^{\mathrm{reg}}=\lim_{\eta \to 0^{+}}\int_{0}^{\infty }dt\;\frac{dJ(t)}{dt}\;e^{-\eta t},$$

where the regularization parameter η resembles the formal convergence parameter in the definition of the Green’s function in terms of the time evolution operator

(20) $$G\left(E^{+}\right)=\lim_{\eta \to 0^{+}}\left(-i\right)\int_{0}^{\infty }dt\;e^{i(E+i\eta -H)t},$$

and is chosen in a similar way as the absorbing potential used in quantum scattering calculations [39,40,41,42]: large enough to accelerate the convergence but still sufficiently small in order not to affect the correct result. This regularization scheme was used in our previous work [27] to mainly compensate for the finite-mode representation of the bath. In this work, however, the numbers of bath modes are sufficiently large to render this effect unimportant. On the other hand, we found oscillations in J(t) due to the Monte Carlo integration of an oscillatory integrand, which is indicated from the observation that the amplitude of the oscillations decreases roughly versus the inverse of the square root of the statistical samples/initial wave functions. We could average out such oscillations by significantly increasing the numbers of samples, which would be too expensive. Instead, we have used time averaging, which is an effective approach for reducing such oscillations in J(t). Thereby, the time-averaged $J(t>t_{0})$ is defined as

(21) $$J^{\mathrm{avg}}\left(t>t_{0}\right)=\frac{1}{t-t_{0}}\int_{t_{0}}^{t}d\tau \;J\left(\tau \right)d\tau ,$$

where $t_{0}$ is the cutoff time when the time averaging starts. We found this approach simpler to implement and it allows us to achieve convergence with a reasonable number of statistical samples. The performance will be discussed in the result section.

### 2.3. Multilayer Multiconfiguration Time-Dependent Hartree Theory

The simulation of the heat current in Equations (15)–(18) involves the evaluation of a quantum mechanical trace and real time propagation for each wave function. The quantum mechanical trace for evaluating the physical observables has the general form

(22) $$\langle A\left(t\right)\rangle =\frac{1}{\mathrm{tr}[\rho ]}\mathrm{tr}\left[\rho \;e^{iHt}Ae^{-iHt}\right].$$

In case that the initial density matrix ρ can be diagonalized, i.e.,

(23) $$\rho =\sum_{N}p_{N}|\Psi_{N}\rangle \langle \Psi_{N}|,$$

Equation (22) reduces to a simple summation

(24) $$\langle A\left(t\right)\rangle =\frac{1}{\sum_{N}p_{N}}\sum_{N}p_{N}\langle \Psi_{N}|e^{iHt}Ae^{-iHt}|\Psi_{N}\rangle ,$$

which can be carried out by Monte Carlo importance sampling methods [29,36,37,38]. On the other hand, when ρ cannot be trivially diagonalized, more sophisticated methods are available to cast Equation (22) into a sum of wave functions with proper weight [37,38]. In this work, each result is obtained from averaging over a few hundred to a few thousand samples/wave functions.

The time evolution for each wave function of the sample, $|\Psi_{N}\left(t\right)\rangle =e^{-iHt}|\Psi_{N}\rangle$, is achieved by employing the multilayer multiconfiguration time-dependent Hartree (ML-MCTDH) theory [29]. ML-MCTDH is a variational method to propagate a wave function of a large system with many degrees of freedom. In this approach a recursive, layered expansion is used to represent a wave function |Ψ(t)〉

(25a) $$|\Psi \left(t\right)\rangle =\sum_{j_{1}}\sum_{j_{2}}\ldots \sum_{j_{p}}A_{j_{1}j_{2}\ldots j_{p}}\left(t\right)\prod_{\kappa =1}^{p}|\varphi_{j_{\kappa }}^{\left(\kappa \right)}\left(t\right)\rangle ,$$

(25b) $$|\varphi_{j_{\kappa }}^{\left(\kappa \right)}\left(t\right)\rangle =\sum_{i_{1}}\sum_{i_{2}}\ldots \sum_{i_{Q(\kappa )}}B_{i_{1}i_{2}\ldots i_{Q(\kappa )}}^{\kappa ,j_{\kappa }}\left(t\right)\prod_{q=1}^{Q(\kappa )}|v_{i_{q}}^{\left(\kappa ,q\right)}\left(t\right)\rangle ,$$

(25c) $$|v_{i_{q}}^{\left(\kappa ,q\right)}\left(t\right)\rangle =\sum_{\alpha_{1}}\sum_{\alpha_{2}}\ldots \sum_{\alpha_{M(\kappa ,q)}}C_{\alpha_{1}\alpha_{2}\ldots \alpha_{M(\kappa ,q)}}^{\kappa ,q,i_{q}}\left(t\right)\prod_{\gamma =1}^{M(\kappa ,q)}|\xi_{\alpha_{\gamma }}^{\kappa ,q,\gamma }\left(t\right)\rangle ,$$

…

where $A_{j_{1}j_{2}\ldots j_{p}}\left(t\right)$, $B_{i_{1}i_{2}\ldots i_{Q(\kappa )}}^{\kappa ,j_{\kappa }}\left(t\right)$, $C_{\alpha_{1}\alpha_{2}\ldots \alpha_{M(\kappa ,q)}}^{\kappa ,q,i_{q}}\left(t\right)$ and so on are the expansion coefficients for the first, second, third,..., layers, respectively; $|\varphi_{j_{\kappa }}^{\left(\kappa \right)}\left(t\right)\rangle$, $|v_{i_{q}}^{\left(\kappa ,q\right)}\left(t\right)\rangle$, $|\xi_{\alpha_{\gamma }}^{\kappa ,q,\gamma }\left(t\right)\rangle$,..., are the single particle functions (SPFs) for the first, second, third,..., layers. The multilayer hierarchy terminates at the bottom level by expressing the SPFs in this layer in terms of (contracted) primitive basis functions. The variational parameters within the ML-MCTDH framework are dynamically optimized through the use of Dirac–Frenkel variational principle [43]

(25d) $$\langle \delta \Psi \left(t\right)|i\frac{\partial }{\partial t}-\hat{H}|\Psi \left(t\right)\rangle =0,$$

which results in a set of coupled, nonlinear differential equations [28,29,44,45,46]. The equations of motion can be effectively propagated using a singular value decomposition (SVD)-based algorithm [47,48]. The ML-MCTDH theory has been generalized to treat identical particles explicitly by employing the second quantized representation [49]. A review of this topic can be found in a recent publication [50]. Furthermore, ML-MCTDH can also be used for calculating energy eigenstates [34,35] or equilibrium reduced density matrices [51].

The form of the ML-MCTDH wave function in Equation (25) has also received much attention recently in applied mathematics [52,53,54]. The original single-layer MCTDH [55,56,57,58] obeys the so-called Tucker form [59] of tensor decomposition. ML-MCTDH [28,29] is then naturally called the hierarchical Tucker (H-Tucker) form and is in fact acknowledged as the first occurrence of the H-Tucker tensor format [52,53,54]. Sometimes it is also called the tree tensor network, although this term or the H-Tucker format usually refers to a binary tree branching structure [53]. Most times a balanced tree is constructed for an ML-MCTDH wave function. For a binary-splitting tree, an n−layer representation holds $2^{n}$ bottom layer SP groups (in our accounting the root node wave function |Ψ(t)〉 does not count as a layer, the first layer comes from the next level). There is also a special skewed tree structure called the tensor train format in mathematics and the matrix-product states in physics [54].

In this work, we adopted a semi-binary tree structure. There are three SP nodes in the first layer, one for the two-level system, one for the left bath, and another one for the right bath. The tree node for the two-level group terminates at the first layer, whereas each node for the left- and right-bath generates two nodes at the next level (the second layer). This binary branching continues until all the bath degrees freedom can be included in the bottom layer. We have used mode combination and basis function contraction [36], under which each bottom-layer SP group may hold up to five bath degrees of freedom. As a result, we have used 6–10 layers in this work to study heat transport where each bath is discretized by a few hundred to a thousand modes. The choice of this semi-binary tree is for convenience and is due to the simplicity of the bath spectral density. For a model with many intramolecular modes, a more complex tree structure may be used [60].

## 3. Results and Discussion

In this work, we focus on studying heat transport dynamics of the spin-boson model at low temperatures: the left bath has zero temperature $T_{L}=0$ whereas the temperature of the right bath is a physical parameter. Other physical parameters include the reorganization energy $\lambda =2\alpha \omega_{c}$ and the characteristic frequency $\omega_{c}$ in Equation (10), chosen to be the same for the two baths, as well as the energy spacing ϵ in Equation (7) for the two-level system. The relative error for the simulations were controlled to be less than 10% based on estimates from repeated ML-MCTDH convergence tests by varying the numbers of primitive basis functions, SPFs, bath degrees of freedom, and statistical samples. Some of these are discussed below. In a typical production run, a total of 1000–2000 bath (left + right) modes were required. This number is greater than that used in the previous work [27], primarily due to the low temperatures. This is because a large density of states is needed to model the condensed phase environment within a finite time of simulation. At lower temperature this translates to more modes. For all the bath modes the number of primitive basis functions ranges from three (for high-frequency modes) to a few tens (for low-frequency modes). A total of 6–10 layers were used in ML-MCTDH simulations. Overall, it appears that the statistical uncertainty is the bottleneck of the calculational error.

**Figure 1:**
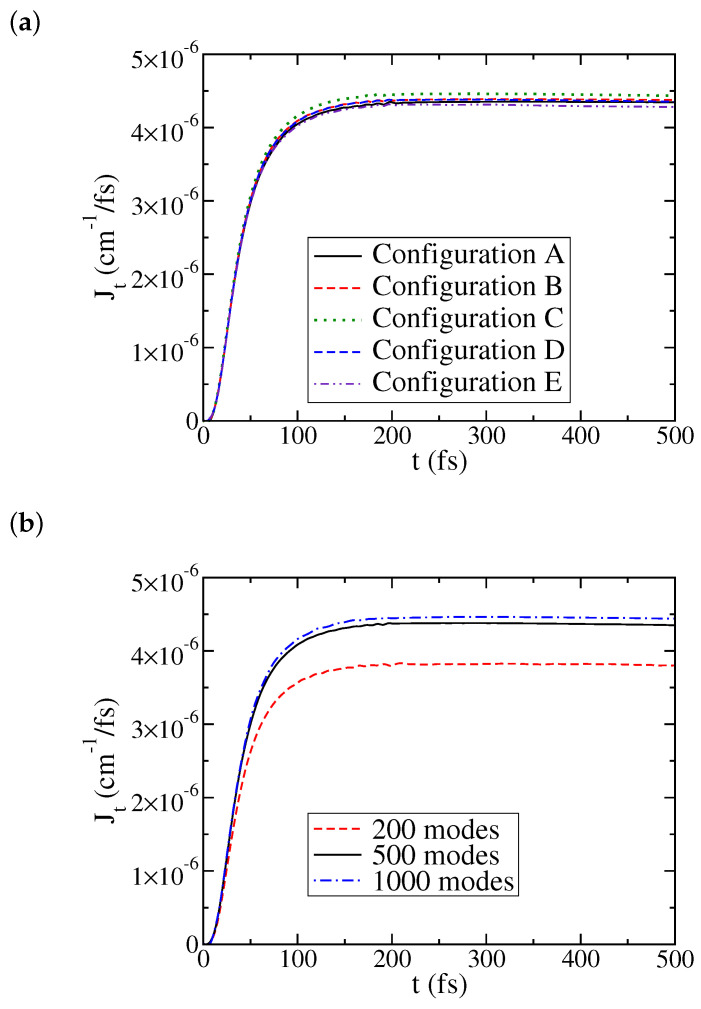
Convergence of J(t) for the set of physical parameters: λ=500 cm${}^{-1}$, $\omega_{c}=500$ cm${}^{-1}$, ϵ=20 cm${}^{-1}$, $T_{L}=0$, $T_{R}=5$ K, and versus: (**a**) different configuration settings with 500 modes for each bath, where Configuration A: 20×4 and 10×3 (meaning 20 single particle functions (SPFs) for each SP group of the top four layers, and 10 SPFs for each SP group of the bottom three layers); Configuration B: 20×3 and 10×4; Configuration C: 16×4 and 8×3; Configuration D: 16×3 and 8×4; Configuration E: 16×2 and 8×5; and (**b**) different numbers of bath modes for each bath.

Figure 1 shows the convergence of the time-dependent heat current versus two set of simulation parameters, different configuration space settings and different numbers of the bath modes. The physical parameters are as follows: the reorganization energy λ=500 cm${}^{-1}$ and the characteristic frequency $\omega_{c}=500$ cm${}^{-1}$ (the same for the two baths), the system energy spacing ϵ=20 cm${}^{-1}$, and the temperatures are $T_{L}=0$ and $T_{R}=5$ K. Figure 1a shows a typical variation of J(t) versus configuration settings when the configuration space is large enough. As mentioned earlier, we adopted a semi-binary tree structure. There are three SP nodes at the first layer, one for the two-level system, one for the left bath, and another one for the right bath. Each tree node for the left- and right-bath generates two nodes at the next level and so on until all the bath degrees freedom can be included in the bottom layer. In this case, there are 500 modes for each bath, and seven layers are used in the simulation. The results are within a few percent of each other, so any setting will do. For most of the results shown in this paper, we used settings similar to configuration C, that is, 16 SPFs (256 configurations under the link node) for the top half number of layers, and 8 SPFs (64 configurations under the link node) for the bottom half layers. It should be noted that although the steady state (averaged) current is converged to within a few percent, this configuration set may not be sufficient for some other purposes. For example, it does not produce a perfect plateau, but a line with a small slope. Using a larger configuration set removes this artifact but at a considerably high cost.

Figure 1b shows the necessity of using a sufficient number of bath modes to obtain accurate heat current. With 200 modes for each bath, both the transient and the steady-state currents have relatively large deviations from the converged results. For results discussed in this paper, we used 500–1000 modes for each bath. The resulting error in J(t) is estimated to be a few percent.

**Figure 2:**
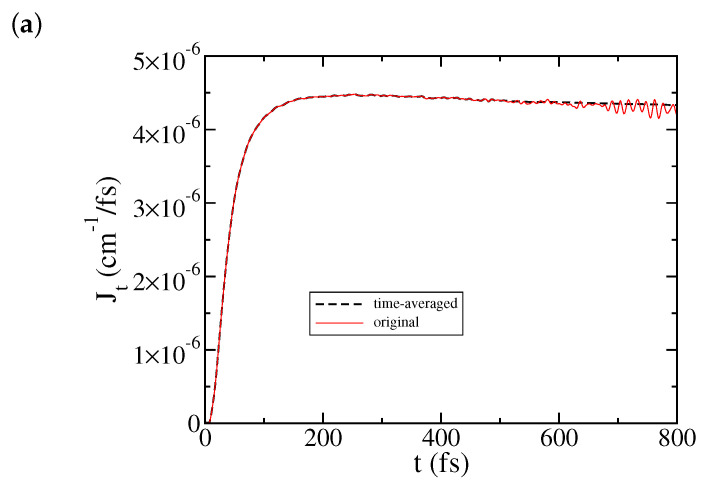

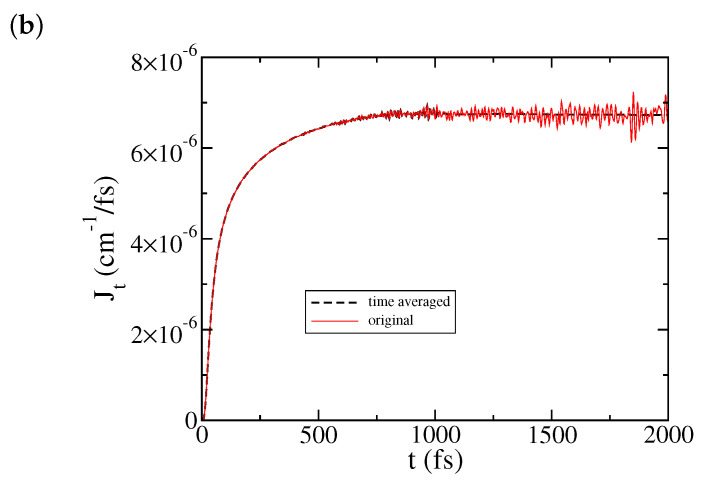
Effect of time averaging on J(t): (**a**) for the same set of physical parameters as in Figure 1, with the starting time $t_{0}=500$ fs in Equation (21); (**b**) for almost the same set of physical parameters as in Figure 1 except $\omega_{c}=550$ cm${}^{-1}$, with the starting time $t_{0}=1000$ fs in Equation (21).

Next we show the effect of time averaging in Equation (21) on J(t). Figure 2a illustrates J(t) for the same set of physical parameters as in Figure 1 where 1000 modes were used for each bath in the simulation. The steady-state plateau for the heat current is established in a relatively short time, so the time averaging can start relatively early, any time between 200 and 500 fs (here we use $t_{0}=500$ fs) in Equation (21). As shown in Figure 2a, the time-averaged J(t) removes the small oscillations at longer time, and gives a more stable value of the steady-state heat current. In this case, time averaging was not really necessary if one is only interested in the steady-state current already established earlier. On the other hand, Figure 2b illustrates a situation where J(t) establishes a plateau in a much later time. In this case, longer time propagation was needed with J(t) displaying larger amplitude oscillations. The starting time for time averaging is later, at $t_{0}=1000$ fs in Equation (21). Figure 2b shows that time averaging works quite well for providing a sensible steady-state current. Thus, for the results discussed below the time-averaged J(t) will be used.

**Figure 3:**
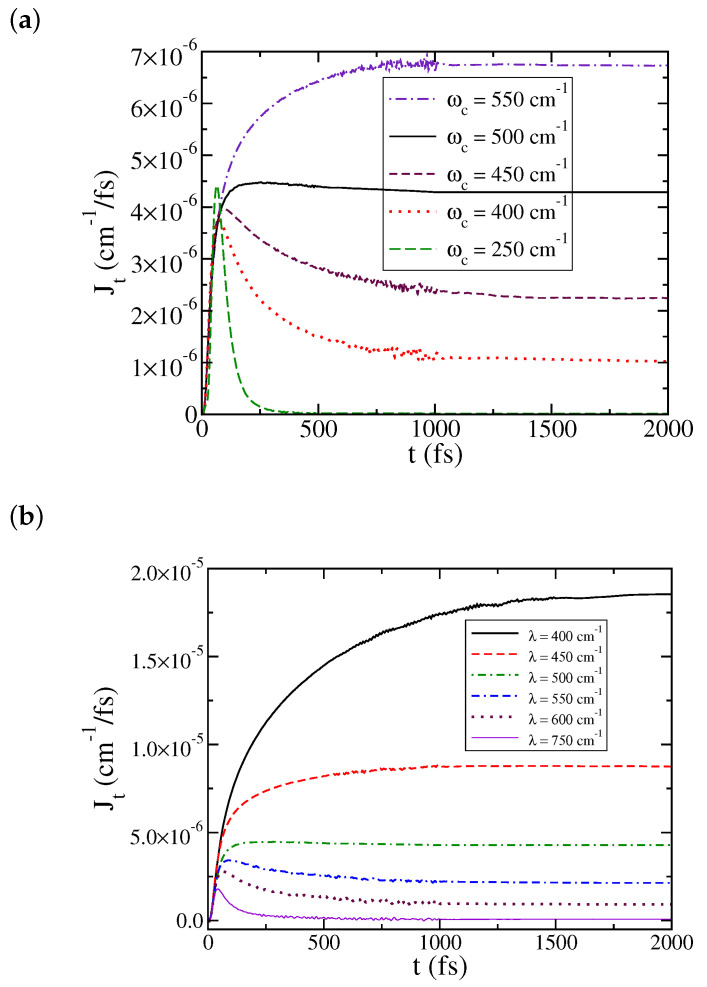
Dependence of J(t) on: (**a**) the characteristic frequency of the bath, $\omega_{c}$ and (**b**) the reorganization energy of the bath, λ. The remaining physical parameters are the same as in Figure 1.

We now discuss the dependence of the heat current on some physical parameters. Figure 3 illustrates the dynamics of J(t) with respect to two parameters, the characteristic frequency of the bath $\omega_{c}$ and the system–bath coupling strength λ (the bath reorganization energy), chosen to be the same for the left and the right bath. The remaining physical parameters are the same as in Figure 1, and 1000 modes are used to represent each bath. The dependence on the bath characteristic frequency $\omega_{c}$ is quite pronounced. As shown in Figure 3a, the steady state current decreases as $\omega_{c}$ decreases, and almost diminishes at $\omega_{c}=250$ cm${}^{-1}$. While our result reveals that a transport blockade occurs at $\omega_{c}<250$ cm${}^{-1}$ for this set of physical parameters, it does not mean that along the opposite direction the heat current keeps increasing with respect to characteristic frequency $\omega_{c}$. Instead, for a larger $\omega_{c}$, J(t) does not reach a well-defined plateau within a simulation time of 4 ps, suggesting long transient coherent behavior.

In a previous study [27], we have found a turnover behavior of the heat current with respect to the system–bath coupling strength λ that resembles the Kramers turnover for the rate constant [61,62]. As shown in Figure 3b, this turnover behavior does not show up with the current set of parameters and at low temperatures. A similar observation has been made for the rate constants at low temperatures [37]. Figure 3b shows the ML-MCTDH simulated steady-state heat current monotonically decreases when the coupling strength λ increases, and is essentially blocked as the coupling reaches a certain level. Again, this does not mean that along another direction the heat current continuously increases as λ decreases. For a small λ, J(t) exhibits long transient coherent behavior and does not reach a well-defined plateau within the simulation time.

**Figure 4:**
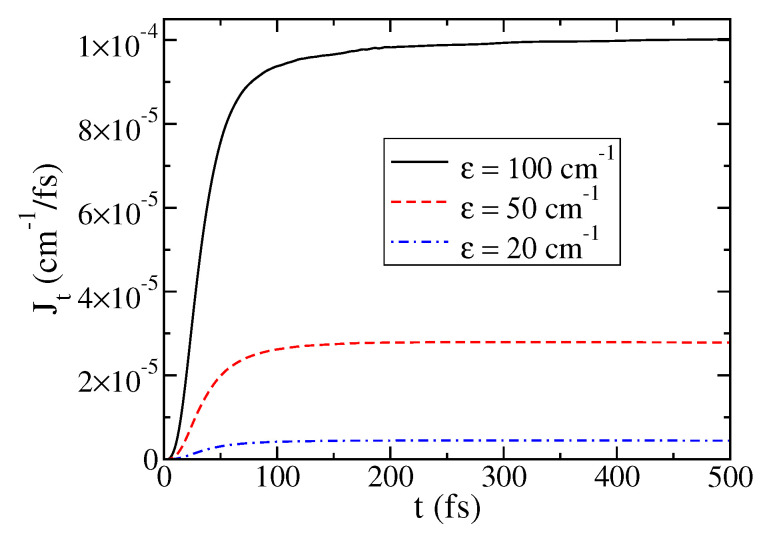
Dependence of J(t) on the the energy spacing of the two-level system ϵ. The remaining physical parameters are the same as in Figure 1.

Figure 4 illustrates the dynamics of J(t) versus the energy spacing of the two-level system ϵ. The remaining physical parameters are the same as in Figure 1, and 1000 modes are used to represent each bath. This dependence on ϵ is what one would expect from a Fermi golden rule approximation treating ϵ as the perturbation parameter, i.e., $J\propto \epsilon^{2}$, but not from the Redfield theory where the system–bath coupling is the perturbation parameter. Given the value of ϵ=20 cm ${}^{-1}$, it is reasonable to assume that the golden rule approach is more appropriate.

**Figure 5:**
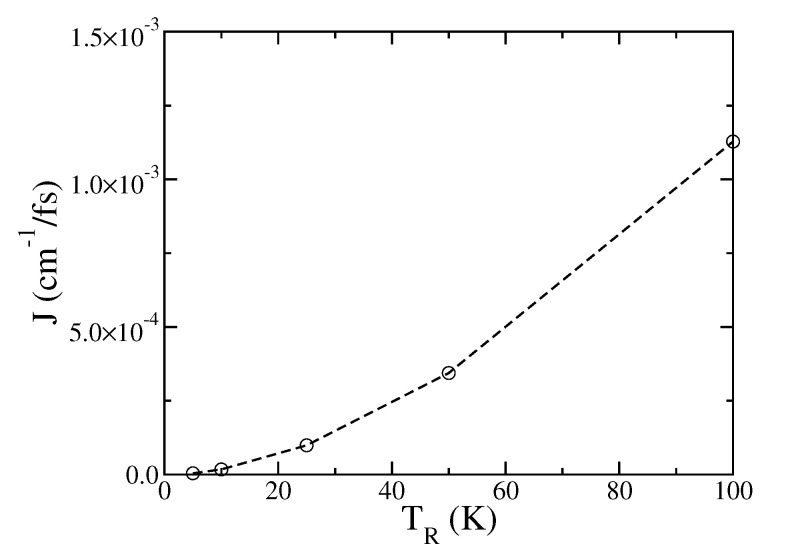
Dependence of the steady-state heat current *J* on the temperature of the right bath, $T_{R}$. The remaining physical parameters are the same as in Figure 1. The line serves as a guide to the eye.

The last physical parameter is illustrated in Figure 5, which shows the dependence of the steady-state heat current *J* on the temperature of the right bath, $T_{R}$, while the left bath is at zero temperature and the remaining physical parameters are the same as in Figure 1. As expected, the heat current increases as $T_{R}$ increases.

**Figure 6:**
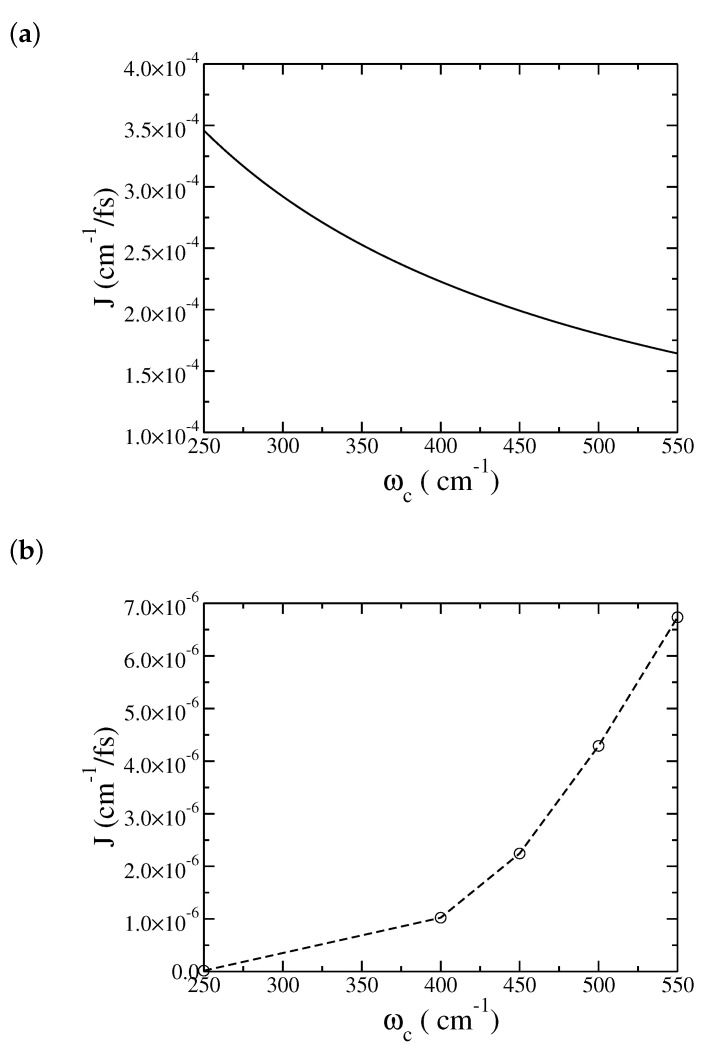
Dependence of the steady-state heat current *J* on the characteristic frequency of the bath $\omega_{c}$: (**a**) Redfield results, and (**b**) multilayer multiconfiguration time-dependent Hartree (ML-MCTDH) results. The remaining physical parameters are the same as in Figure 1.

One may be curious about the performance of Redfield theory that was initially used in the study of heat transport in the spin-boson model [7,8,9,16,27]. The following discussions compare the Redfield approximation with our ML-MCTDH simulations. First shown in Figure 6 is the dependence of the heat current on the characteristic frequency. Not only are the Redfield currents much bigger than our simulation results, but also the trend is different: while our ML-MCTDH simulation predicts an increase in the heat current as the characteristic frequency increases, Redfield theory predicts the opposite—that *J* decreases upon increasing $\omega_{c}$. The disagreement can be rationalized by noting the value of the system–bath coupling, λ=500 cm${}^{-1}$, which is not in the weak coupling regime that Redfield theory operates. In addition, the steady-state Redfield theory used here [7,8,9] assumes that heat transfer is dominated by resonance energy transmission and that dephasing processes are fast, neither is satisfied for the set of physical parameters in the low-temperature heat transport processes studied here.

**Figure 7:**
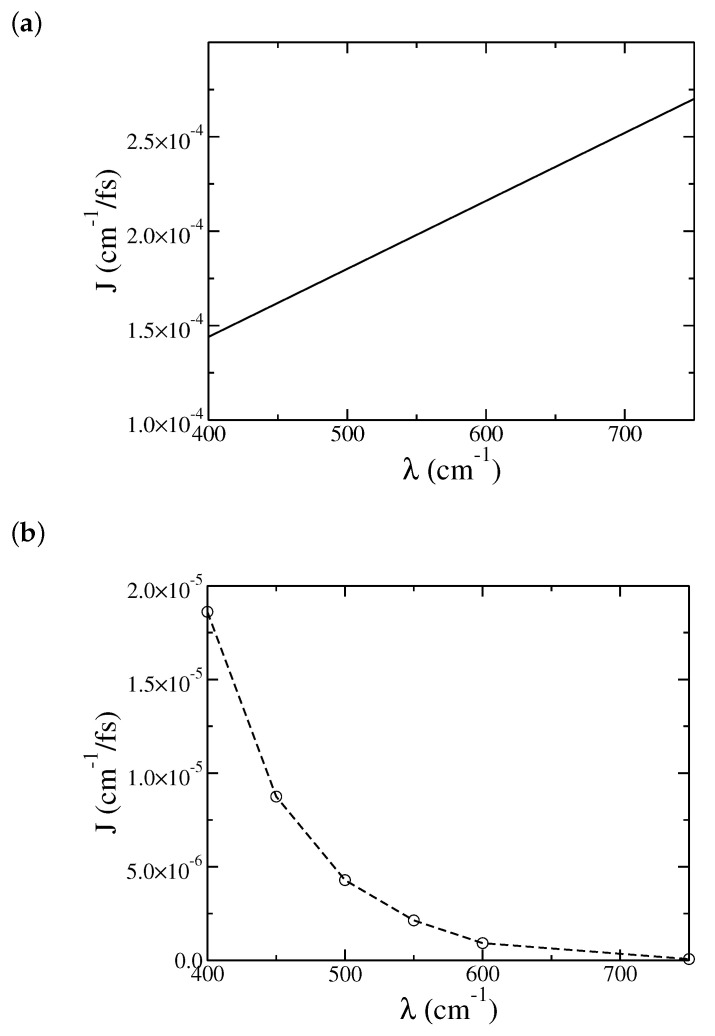
Dependence of the steady-state heat current *J* on the reorganization energies of the bath, λ: (**a**) Redfield results, and (**b**) ML-MCTDH results. The remaining physical parameters are the same as in Figure 1.

Figure 7 shows both Redfield approximation (Figure 7a) and our ML-MCTDH simulations (Figure 7b) where the system–bath coupling λ is varied. Again, Redfield theory gives too large current values, and also an opposite trend for *J* versus λ in comparison with the simulation results. As discussed in a previous work [16], Redfield theory is no long a good approximation as the system–bath coupling increases. A turnover behavior of the heat current with respect to the system–bath coupling strength will be observed [27] and is best described by some improved theories [16,17,22]. On the other hand, Figure 7b does not show a turnover behavior at low temperatures. As discussed earlier, this is because the steady-state current is not well defined (at low temperatures) for a weak system–bath coupling λ, where J(t) exhibits long time coherent behavior instead of reaching a well-defined plateau. In this weak coupling regime, it is an interesting future task to study transient dynamics and possibly how to use this information to extract the steady-state current at very long time.

**Figure 8:**
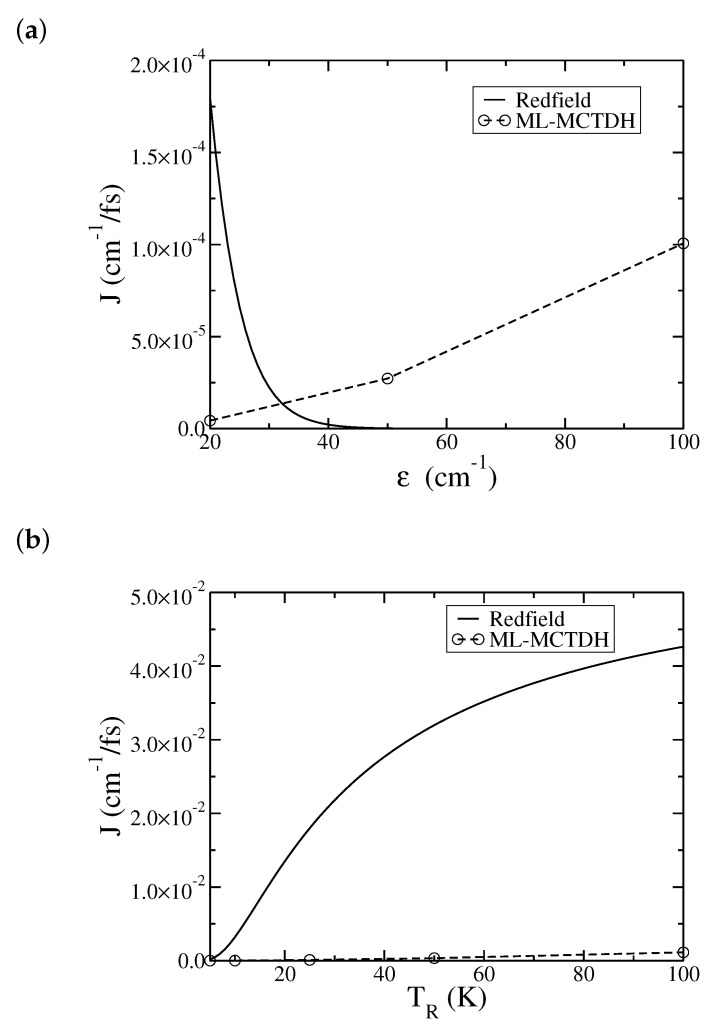
Dependence of the steady-state heat current *J* on (**a**) the energy spacing of the two-level system ϵ and (**b**) the temperature of the right bath, $T_{R}$. The solid line is from Redfield theory and the dashed line with circle is from ML-MCTDH simulation (the dashed line serves as a guide to the eye). The remaining physical parameters are the same as in Figure 1.

For most of the parameter sets considered in the paper, the ML-MCTDH simulated heat currents are almost two orders of magnitude smaller than the Redfield theory results. Thus, the comparisons were made in separate plots in Figure 6 and Figure 7. Our final comparisons between Redfield theory and the ML-MCTDH simulations are illustrated in Figure 8, in which the results are overlaid. Figure 8a shows the dependence of the steady-state current *J* on the level spacing ϵ of the two-level system. Redfield theory shows a quick drop in the current as ϵ increases, which is a manifestation of low temperatures in the Redfield expression. On the other hand, the ML-MCTDH simulation shows an approximate quadratic increase for the heat current as ϵ increases, $J\propto \epsilon^{2}$, consistent with the golden rule approximation where ϵ is the perturbation parameter. This behavior will eventually breakdown when ϵ becomes too large, and will be an interesting subject for the future. Finally, Figure 8b shows the temperature dependence of the steady-state current. In this case, Redfield theory predicts the correct qualitative trend, that *J* increases as *T* increases. Its values are, however, too big as compared with the simulation results.

## 4. Conclusions

In this paper, we have employed the ML-MCTDH theory to simulate heat transport processes in a spin-boson junction model at low temperatures. Compared with our previous work [27], more bath modes are needed to represent the continuum at low temperatures, thus requiring more layers for the tensor contraction in ML-MCTDH. Time averaging is also a useful technique for obtaining the steady-state current. Dependence of the heat current has been investigated versus several physical parameters, i.e., the energy spacing for the two-level system, the characteristic frequency of the bath, the system–bath coupling strength, and the temperature difference between the two baths. Comparisons have also been made with the Redfield theory approximation. Interesting behavior at low temperature has been observed, primarily of strong quantum origin.

The ML-MCTDH benchmark results will be helpful for developing physical theories to study heat transport processes at low temperatures. This is of both theoretical interest and practical use for designing small devices under extreme conditions. A particularly interesting question, which is beyond this work but worth studying in the future, is whether a steady-state heat transport can ever be established at low temperatures. Under such conditions, the quantum coherence effect may be prominent. Even a steady-state current can be reached at infinite time in theory, finite-time transient dynamics of J(t) may be dominant for determining practical heat transport.
